# Fermentation Biotechnology Applied to Cereal Industry By-Products: Nutritional and Functional Insights

**DOI:** 10.3389/fnut.2019.00042

**Published:** 2019-04-12

**Authors:** Michela Verni, Carlo Giuseppe Rizzello, Rossana Coda

**Affiliations:** ^1^Department of Soil, Plant and Food Science, University of Bari Aldo Moro, Bari, Italy; ^2^Department of Food and Environmental Science, University of Helsinki, Helsinki, Finland

**Keywords:** cereal by-product, fermentation, yeast, lactic acid bacteria, bioactive compounds, antioxidant activity, anticancer

## Abstract

Cereals are one of the major food sources in human diet and a large quantity of by-products is generated throughout their processing chain. These by-products mostly consist of the germ and outer layers (bran), deriving from dry and wet milling of grains, brewers' spent grain originating from brewing industry, or others originating during bread-making and starch production. Cereal industry by-products are rich in nutrients, but still they end up as feed, fuel, substrates for biorefinery, or waste. The above uses, however, only provide a partial recycle. Although cereal processing industry side streams can potentially provide essential compounds for the diet, their use in food production is limited by their challenging technological properties. For this reason, the development of innovative biotechnologies is essential to upgrade these by-products, potentially leading to the design of novel and commercially competitive functional foods. Fermentation has been proven as a very feasible option to enhance the technological, sensory, and especially nutritional and functional features of the cereal industry by-products. Through the increase of minerals, phenolics and vitamins bioavailability, proteins digestibility, and the degradation of antinutritional compounds as phytic acid, fermentation can lead to improved nutritional quality of the matrix. In some cases, more compelling benefits have been discovered, such as the synthesis of bioactive compounds acting as antimicrobial, antitumoral, antioxidant agents. When used for baked-goods manufacturing, fermented cereal by-products have enhanced their nutritional profile. The key factor of a successful use of cereal by-products in food applications is the use of a proper bioprocessing technology, including fermentation with selected starters. In the journey toward a more efficient food chain, biotechnological approaches for the valorization of agricultural side streams can be considered a very valuable help.

## Introduction: Overview of the Fate of Cereal Industry By-Products

Cereals are the edible seeds of the grass family of *Poaceae*, also known as *Gramineae*, and their cultivation dates to thousands of years ago. Wheat, maize, rice, barley, sorghum, millet, oat, and rye are the cereals most important on a global scale (1). Among them, wheat and rice represent the dominant crops, in Western and Asian countries, respectively (2, 3). Cereals are one of the most important food sources for human consumption, with a production of more than 2 billion tons/year. However, unfortunately, roughly 30% of this amount is wasted or lost due to several reasons (2, 4). Overall, food losses include all the edible parts discarded during the supply chain, while food wastes are residues of high organic load, removed during raw materials processing to foodstuff (5). In developing countries, substantial food losses occur during agricultural production, whereas in industrialized countries losses also include processed products during the distribution and consumption stages (5). Considering the unused food matrix as waste does not enforce the possibility of re-utilizing it in the food chain. For this reason, the use of the term “by-product” is increasing and identifies those wastes that become substrates for the recapture of functional compounds and the development of new products with a market value (6).

In cereals processing, the two major by-products obtained during the traditional milling procedures are bran and germ. The initial purpose of milling was mostly to grind the grain, successively, the separation of the starchy endosperm from the outer layers (dry milling) became more important. The main reason for germ and bran removal is that, despite being rich in vitamins, minerals and dietary fiber, they adversely affect the processing properties of flours and this is one of the main reasons why the majority of cereal foods consumed are made of refined flour (7, 8). However, refined flour is then lacking many compounds important for nutrition (8). Wet milling, on the other hand, is mainly used to produce starch and gluten, separating them from germ, bran and a precipitate solid fraction (9).

**Figure 1:**
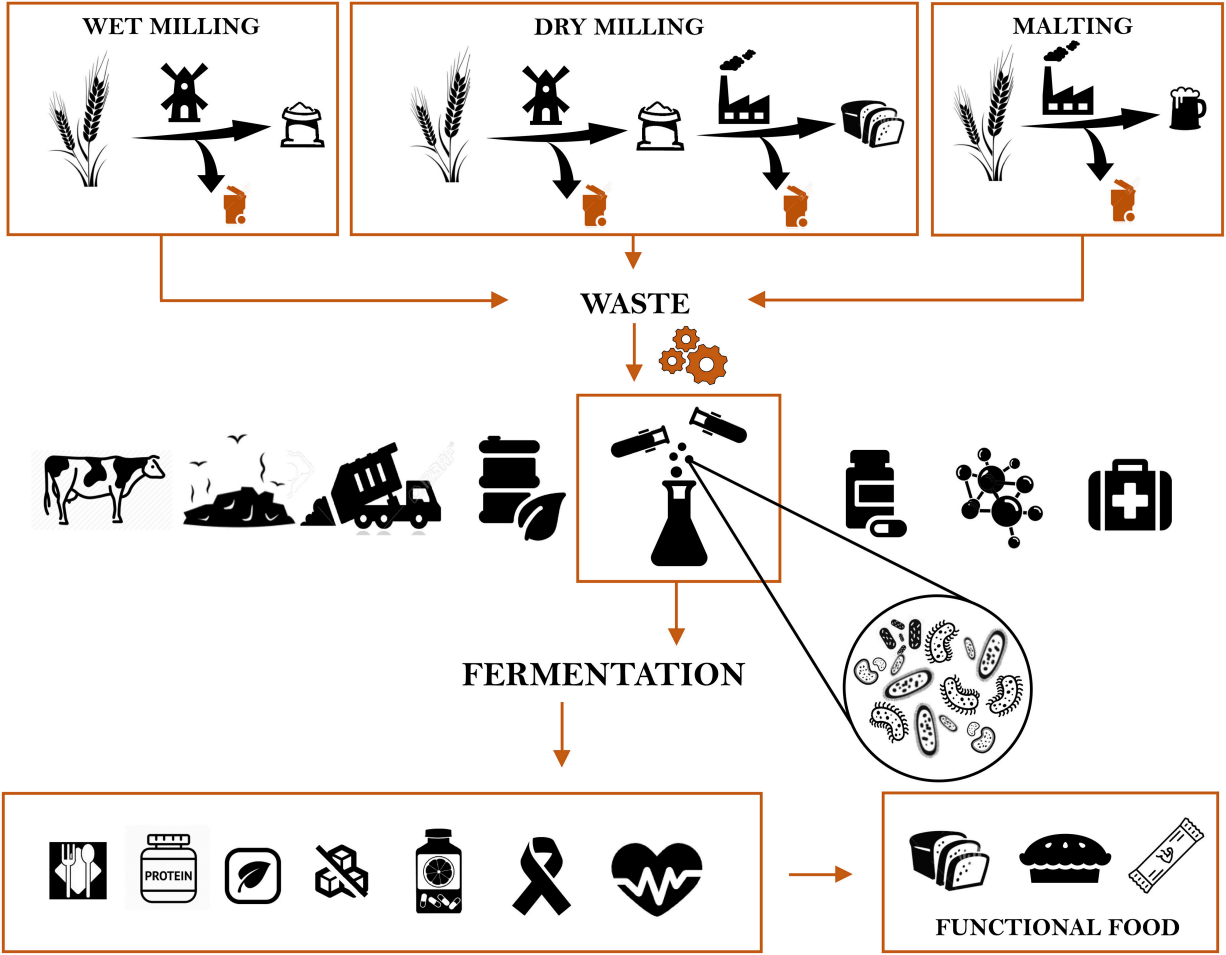
The cereal industry by-products chain, from generation to the disposal, and their novel applications for improving nutritional and functional features of food.

The employment cycle of these by-products can follow different paths (Figure 1). The most common way of disposal is to use them as feed or for compost. However, to alleviate the environmental and economic burden of such losses, different approaches have been explored (10). One of them is biorefinery to produce biofuels such as ethanol (11). Ethanol is often produced from the cellulosic fractions of cereal bran, especially from maize. It is estimated that 4% of the global grains are used for ethanol production (12). Besides ethanol the production of chemical compounds such as lactic acid is also pursued (13). Lactic acid is traditionally applied in food industry as well as in pharmaceutical, textile and chemical industry. It can be obtained from an extensive range of carbon sources, of which cereal industry by-products such as maize cob, wheat bran, brewer's spent grains are very rich (14). Whereas, maize, rice and wheat bran are often used to produce phytic acid. After phytic acid extraction, the resulting by-product is deprived of an antinutritional factor, making it a more valuable ingredient in animal feed (12). Insoluble dietary fibers, fructans, antioxidants, and many other bioactive compounds are extracted from cereal by-products and used in food manufacturing (10). The oil obtained from wheat germ finds wide application in vitamin production, and cosmetic industry as well as in food, feed and as insect biological control agent, while the defatted wheat germ and wheat germ proteins are used as ingredients for several food products (15).

A more recent approach involves the generation, from rice and wheat bran, husk and straw, of nanoparticles exhibiting antibacterial activity or the production, through microbial fermentation, of biodegradable plastics (10). Cereal by-products are considered an ideal candidate to produce commercially important enzymes, due to their richness in nutrients but also to their low costs and wide availability as cultivation substrates. The use of cereal by-products is considered one of the most effective means in producing high value compounds (10).

Besides enzymes production or bioplastics synthesis, microbial fermentation is a very efficient way to improve the nutritional and functional properties of cereal by-products to implement their use in food production. Fermentation, alone ore coupled with technological or biotechnological processing techniques, offers a variety of tools to modify cereal matrices. During fermentation, both endogenous and bacterial enzymes are able to modify the grain constituents affecting the structure, bioactivity, and nutrient bioavailability (16). Since fermentation with either lactic acid bacteria, yeasts or fungi is extensively employed to produce cereal-based foods with enhanced health properties (17, 18), its application to cereal by-products could, to a wider extent, improve the overall eco-sustainability of the food system, providing a suitable alternative to reduce malnutrition and hunger (19). In light of the above considerations, this review aims at providing a comprehensive overview of all the nutritional and functional positive outcomes deriving from fermentation of cereal industry waste and by-products.

## Fermentation of the Milling By-Products

### Wheat Bran

The multiple outer layers of wheat (outer and inner pericarp, seed coat, and nucellar epidermis) are commonly referred to as bran (20). During conventional wheat roller milling, most of the endosperm is separated and further ground to wheat flour. Therefore, bran, together with the aleurone layer and remnants of endosperm, becomes a milling by-product. Different types of bran (i.e., coarse bran or regular bran, coarse weatings or fine bran, fine weatings or middlings or shorts, and low-grade flour or “red dog”) can be distinguished depending on the particle size and the endosperm content (21). The most abundant polysaccharides of the bran layers, arabinoxylans and β-glucans, have a role in lowering the risk of type II diabetes and colorectal cancer as well as cardiovascular and diverticular diseases (8). However, bioactive compounds such as dietary fibers and phenolic acids are trapped in the cell wall structures resisting conventional milling and thus having low bioaccessibility (22). Thereby, new milling techniques, enzymatic treatments and fermentation processes targeting the structure of bran, have been studied with the aim of enhancing its nutritional potential (23).

Over the last years, the interest of the scientific community toward wheat bran fermentation, alone or combined with other approaches, markedly increased. Fermentation with two selected microbial strains (*Lactobacillus brevis* E95612 and *Kazachstania exigua* C81116) combined with hydrolytic enzymes, mainly xylanase, endoglucanase, and β-glucanase was employed to obtain bran with higher nutritional quality than the native one (24). Bioprocessing influenced the microstructure of the bran, causing an extensive breakdown of the cell wall structures entailing an increase of the solubility of arabinoxylans, more than 11-fold compared to the native bran (24). These results confirmed those obtained in a previously study (6) in which yeast fermentation of wheat bran led to a 66% increase in soluble arabinoxylans. Wheat bran was also fermented following a traditional fermentation process consisting of a back-slopping procedure of daily refreshments until a stable microbiota of lactic acid bacteria and yeasts was established. After fermentation, an increase up to 30% of soluble dietary fiber was obtained (25). Similar results were observed in a recent study fermenting bran with *Lactobacillus bulgaricus* and *Streptococcus thermophilus* combined with a commercial baker's yeast (26).

Wheat bran is also a rich source of proteins and amino acids, which, although different from those found in the endosperm, still have high biological and nutritional value (27). However, the bioavailability of proteins in bran is limited by multiple factors, (i) the structure of the layers (composed by insoluble and complexed carbohydrates and lignin) and (ii) the high content of antinutritional factors such as phytate which forms insoluble phytate-protein complexes (28). Because of lactic acid bacteria proteolytic activity and endogenous proteases activated by the low pH, increases of peptides and free amino acids concentration, including the functional non-protein γ-aminobutyrric amino acid (GABA), have been reported during fermentation (24, 25, 29). Consequentially, the *in vitro* digestibility of proteins, which gives information on their stability and on how they withstand to digestive processes, increased. The digestible protein fraction can also give information about the quality of the protein itself. It was indeed proven that the biological value, the ratio of essential amino acids, and the nutritional index were higher in fermented bran compared to the native one, and further increase of the above indexes was obtained when enzymes were used in combination with microbial fermentation (24). The use of the starter cultures *L. brevis* E-95612 and *Candida humilis* E-96250, especially when cell-wall-degrading enzymes were added, affected not only the release and the composition of free amino acids, improving protein digestibility, but also enabled the release of phenols (29). Hydroxycinnamic acids are the most common phenolic acids found in wheat bran. In particular, ferulic acid, a structural component of the cell walls of aleurone and pericarp is mainly esterified with arabinoxylans, and therefore has a very low bioaccessibility. The potential health effect of ferulic acid is ascribed to its antioxidant properties. In particular, its ability to inhibit the lipid peroxidation and the oxidation of low-density lipoprotein (the main cholesterol carrier in blood), is greater than other hydroxycinnamic acids (30). Ferulic acid also has anti-inflammatory effects (30). Bioprocessing of wheat bran with baker's yeast and an enzymes mixture containing ferulic acid esterase allowed the increase of free phenolic acids content (31). Spontaneous fermentation carried out by lactic acid bacteria (mainly belonging to the genera *Lactobacillus, Leuconostoc* and *Pediococcus*) and yeasts was also found to be effective on the release of ferulic acid, which increased of 82% (25). The release of ferulic acid occurred also during fungal fermentation. Among the edible mushrooms employed in a previous study, *Hericium erinaceus* was the one that allowed the highest release of ferulic acid (44% higher than the unfermented bran) due to the combined action of cellulase and ferulic acid esterase which were able to decompose wheat bran cell walls (32). An improvement in total phenol content and antioxidant activity toward free radical DPPH (2,2-diphenyl-1-picrylhydrazyl) and ABTS (2,20-azino-di-[3-ethylbenzthiazoline sulphonate]) occurred when wheat bran was fermented for 6 days at 30°C with *Aspergillus oryzae* MTCC 3107 (33). Maximum antioxidant activity was noticed for fermented bran, in both methanol and ethanol extracts, compared to fermented wheat intact kernels and flour (33). The role of oxygen during the fermentation of a liquid wheat bran sourdough was also investigated. The amount of oxygen influenced the microbial community, as well as the metabolite profile of fermented bran. It was indeed observed that anaerobic conditions, in which lactic acid bacteria and endogenous heterotrophic bacteria grew better, induced the conversion of ferulic and caffeic acids into their corresponding derivatives, and increased the amount of sinapic acid. On the contrary, wheat bran fermented in aerobic conditions, which favored yeasts growth, was characterized by the presence of the phenolic compounds dihydroxyphenyl ethanol and hydroxyphenylacetaldehyde. Moreover, a higher amino acids content was found after anaerobic fermentation compared to aerobic one (34).

Minerals and vitamins contained in wheat grains are mostly located in the bran fraction, especially in the aleurone layer. The bioavailability of minerals strongly depends on the content of phytic acid, which is generally very abundant in wheat bran and is considered an antinutritional factor. An improvement in phytase activity was observed when both fermentation and enzymes were used to bioprocess bran (24, 29) and a reduction of phytic acid was reported by several authors (25, 26, 35).

Several B-vitamins, mainly niacin, pantothenic acid, biotin, thiamin and small amount of riboflavin are present in wheat bran (30). Yeast fermentation successfully increased the folate content of wheat bran over 40% (36). In the study conditions, folate synthesis, which varies extensively between *Saccharomyces cerevisiae* strains, was also partially ascribed to the presence of indigenous lactic acid bacteria. The fortification in limiting vitamins was also proposed. For instance, the intake of vitamin B12, also known as cobalamin, is mainly possible through consumption of food from animal origin (37), therefore there is a risk of its deficiency for people consuming limited amount of animal food products. Thus, the fortification of plant-based food with this vitamin through fermentation represents a good strategy. *Propionibacterium freudenreichii* DSM 20271, one of the few microorganisms recognized as vitamin B12 producers, was used to ferment wheat bran. The content of the active form of vitamin B12 in fermented bran increased of about 5 times, and a higher content in riboflavin was also detected, proving that bran can be a potential substrate for vitamins synthesis (38).

The use of milling by-products in food processing entails technological drawbacks, which make their application more challenging. For instance, in wheat bread baking, gluten has a key role in the structure formation and bran addition weakens the gluten network structure, therefore affecting the gas-holding capacity of doughs. As a result, volume and elasticity of baked goods decrease (23).

An increase in the phenolic content and therefore in the antioxidant activity of bread containing wheat bran fermented with either yeasts or lactic acid bacteria was reported by few authors (36, 39, 40). Anson et al. (31), fermented wheat bran with baker's yeast and used it to produce a fortified bread having almost 3-fold the amount of free ferulic acid and 8-fold when combined with hydrolytic enzymes treatment. *p*-coumaric and sinapic acids also increased with the processing. When subjected to gastro-intestinal digestion *in vitro*, despite the substantial increase in the bioavailability of phenolic compounds, mostly recovered from the jejunal compartment, only a small part of them was further metabolized in the colon section, especially those that were already partially degraded by the bran fermentation and enzymatic treatments. Colonic metabolites have been found to have anti-inflammatory properties suggesting that bread enriched in fermented wheat bran could show the same properties (31).

Fermented wheat bran was used to prepare a composite wheat-rye bread containing a β-glucan hydrogel. Although the flavonoid content was significantly higher in the experimental bread compared to the control, the concentration of phenolic acids decreased during sourdough fermentation. However, higher radical scavenging activity against both ABTS and DPPH radical were found. The same bread was found to lower the glucose response 120 min after the consumption in a small group of volunteers. Insulin response did not change compared to the control bread (wheat-rye bread), but the authors indicated that an increased amount of fermented wheat bran and β-glucan could further improve the nutritional impact of the wheat bran-rye bread (41).

### Wheat Germ

Wheat germ is a high nutritional value by-product separated during the milling process. It is the primary source of vitamin E in wheat kernel and a rich source of vitamins of the group B, proteins, dietary fiber and minerals (30, 42). Most of the essential amino acids are present in wheat germ proteins at concentrations higher than in the reference egg protein pattern (43, 44). Wheat germ is also rich in unsaturated fatty acids, mainly oleic, linoleic and α-linoleic acids and functional phytochemicals especially flavonoids, sterols, octacosanols, and glutathione (45). However, its consumption is limited by some anti-nutritional factors (raffinose, phytic acid, and wheat germ agglutinin) and by the high lipase and lipoxygenase activity that favor lipid oxidation, negatively affecting the stability of wheat germ (15).

To solve this issue, the effects of sourdough fermentation on wheat germ stabilization were studied. Two lactic acid bacteria (*Lactobacillus plantarum* LB1 and *Lactobacillus rossiae* LB5) isolated from wheat germ were used as starters for sourdough fermentation (46). After 40 days of storage, compared to the raw germ, the fermented one had very low percentage of the aldehydes usually responsible for the rancidity perception, as well as of alcohols, ketones, furanones, and lactones, other volatile compounds occurring in lipid oxidation. The low pH achieved with fermentation was indeed responsible of the lower lipase activity. Fermentation also increased of ca. 50% the concentration of total free amino acids, more specifically Lys, the major limiting amino acid of wheat flour, and GABA were present in fermented wheat germ at the concentration of almost 2 g/kg (46). During sourdough fermentation of wheat germ, the phytase activity increased and an enhanced the bioaccesibility of Ca^++^, Fe^++^, K^+^, Mn^++^, Na^+^, and Zn^++^. Concomitantly, raffinose concentration decreased by 45% and a 33% increase in phenol content occurred, which resulted in higher scavenging activity toward free radical DPPH and ABTS (46). Antioxidant activity in food matrixes is often due to the presence of phenolic compounds; nonetheless, this functional property can also be ascribed to bioactive peptides. Biologically active peptides, often encrypted in the native sequence, can be produced from their protein precursor by digestive enzymes or during food processing (47). The interest toward bioactive peptides from vegetable sources has increased thanks to the recent evidence of their wide potential functional effects (antihypertensive, antioxidant, antitumoral, antiproliferative, hypocholesterolemic, antinflammatory activities) (48). After 48 h of fermentation of a medium composed by 5% of defatted wheat germ, the maximum yield of peptides was obtained. The protein hydrolysate showed high antioxidant activity, determined as scavenging activity on DPPH, hydroxyl, and superoxide radicals (49).

One of the most promising features of fermented wheat germ is represented by the cytotoxic activity toward cancer cell lines. A commercially available wheat germ extract known as Avemar^®^ is obtained by fermentation of wheat germ water-soluble extract with *Saccharomyces cerevisiae*, followed by concentration and drying (50, 51). The anticancer properties of this extract have been shown *in vitro* on various human cancers cell lines (including leukemia, melanoma, breast, colon testicular, head and neck, cervical, ovarian, gastric, thyroid, and brain carcinomas), as well as on the prevention of chemical carcinogenesis, and some autoimmune conditions (50, 52). These features are mainly attributed to two quinones, 2-methoxy benzoquinone and 2,6-dimethoxybenzoquinone which are naturally present in wheat germ as glycosylated and non-physiologically active form. For this purpose, selected strains of lactic acid bacteria possessing high β-glucosidase activity, therefore potentially able to release the two quinones, were used for wheat germ fermentation (53). During 24 h of incubation, the release of the non-glycosylated and physiologically active forms was almost complete. Compared to the control, the concentration of the above bioactive compounds increased up to 6-folds. While no effect was found for the raw wheat germ, the preparation fermented by *Lb. plantarum* LB1 and *Lb. rossiae* LB5 exerted anti-proliferative effect on human tumor cell lines (colon carcinoma and ovarian carcinoma), as showed by *ex vivo* assays (53).

Germ is rarely used for food processing mainly because of the short shelf-life, due to the presence of large amounts of unsaturated fat acids and of hydrolytic and oxidative enzymes (54), as well as to the negative effects on the technological properties of the flour. Fermented wheat germ, having lipase activity lower than raw wheat germ, was incorporated in bread effectively reducing the technological obstacles that prevent its use in baking (55). The concentration of total free amino acids, especially that of lysine, which is limiting in cereals, increased if compared to wheat bread. GABA content increased as well reaching 223 mg/kg. Among the nutritional benefits deriving from wheat germ fermentation and supplementation in bread manufacturing there were improved *in vitro* protein digestibility and decreased phytase activity, all without compromising baking properties (55). In a recent study, a sourdough composed by both wheat germ and bran was used to fortify wheat flour bread (39). In addition to the release of free amino acids and phenolic compounds, which reflected on the antioxidant activity, the fortified bread containing 15% of the above fermented milling by-products had 6.53% of dietary fiber, almost 5% higher than that of wheat flour bread. The *in vitro* and, especially, the *in vivo* glycaemic index were markedly lower in the fortified bread, reaching 36.9%, a value way below the threshold needed for a food product to be considered “low glycaemic index” (39).

### Rye Bran

Rye, one of the most important sources of dietary fiber in European Nordic countries, is often used as whole grain flour in the making of cereal based products. Nonetheless, rye bran is also a by-product of conventional milling and can be used as ingredient to increase food nutritional value (56). Besides fibers, the bran fraction is rich in many other bioactive compounds (phenolic acids, phytosterols, tocopherols), among which alkylresorcinols and steryl ferulates have been studied for their cancer preventive and antioxidant potential (7, 57, 58). The influence of fermentation conditions and type of bran (native or peeled) on the levels of bioactive compounds was studied (59). Bran fractions, deriving from native or peeled grains were fermented with baker's yeast for 6-20 h at temperatures ranging from 20 to 35°C. Fermentation of both peeled and native bran increased free ferulic acid and free total phenolics, indicating increased liberation of bound phenolic compounds from the polymeric rye bran structure. The increase in free phenolics after fermentation was reported to be 90% for native rye bran, and 30% for peeled bran (59). The level of folates increased over 100% with fermentation and the highest level was obtained when higher growth of indigenous lactic acid bacteria occurred, which was strongly dependent on fermentation conditions (higher temperature and longer time). However, the strong acidity was found to be deleterious for cinnamoyl esterases, the enzyme responsible for the release of ferulic acid esterified to the arabinofuranosyl residues (60). For this reason, the highest level of free ferulic acid was obtained in fermentation conditions with a pH value of 6–6.5 (59).

Several authors studied the influence of rye bran bioprocessing on bread nutritional quality. When combining the action of hydrolytic enzymes and baker's yeast fermentation the main change was represented by the degradation of the cell wall structure which consequentially led to an increase in the solubility of dietary fibers, arabinoxylans, proteins, and free phenolics (61). As shown by the *in vitro* colon model, bioprocessing made carbohydrates more fermentable by the microbiota and allowed a higher release of ferulic acid. In a follow up study, the phenolic profile of bread fortified with bioprocessed rye bran was evaluated (62). *p*-cumaric, sinapic, and caffeic acids were detected at concentration 20–30-fold higher than the bread containing native rye bran. Additionally, although a slight alkylresorcinol degradation was observed, benzoxazinoid aglycones increased after the enzymatic bioprocessing. Despite the extensive phenolic acid release caused by the bioprocessing, when breads were subjected to *in vitro* colon model, only subtle differences were observed in the microbial metabolites (63).

Rye bran proved to be an excellent substrate for the synthesis of polyunsaturated fatty acids and β-carotene by fungal solid-state fermentation (64). Four *Mucor* spp. strains selected based on the ability to synthesize γ-linolenic acid (GLA) and β-carotene were used. Compared to oat flakes, barley groats and wheat bran, rye bran fermented with *Mucor circinelloides* CCF-2617 contained the highest content of both compounds, which was further increased by the addition of brewers' spent grain (64).

Rye and wheat bran were used as substrates for the synthesis of exopolysaccharides through lactic acid bacteria fermentation. Fermentation with *Lactobacillus reuteri* in the presence of sucrose enabled high glucan formation within 8 h of incubation of rye bran (65). In a recent study, two strains of *Weissella confusa*, known for the ability to produce significant amounts of dextran, were employed to ferment both wheat and rye bran. Rye bran proved to be an optimal substrate for *in situ* dextran production, reaching concentrations of 2–3% on dry matter (66). The *in situ* synthesis of EPS and oligosaccharides represents a way to obtain a food substrate with increased functional properties. In fact, they may act as prebiotic and have been shown to possess antitumor and immunomodulating activities *in vitro* (67). Additionally, EPS also act as hydrocolloids and can improve the technological properties of material like cereal bran, otherwise characterized by poor structure-forming capacity.

### Rice Germ and Bran

Asian Countries are the major producers of rice, representing 50% of the daily energy supply of the diet of the local population (68). From the commercial white rice, germ and bran are removed because the oils they contain are quickly subjected to rancidity, reducing its shelf-life (7). It is estimated that every year 120,000 tons of rice husks alone are wasted worldwide (10). Rice milling by-products are currently underutilized, since their further exploitation is possible. Rice bran oils and proteins have demonstrated antioxidant properties and chronic disease preventing activity, particularly toward cardiovascular disease and certain cancers (69–71). However, the content of these bioactive compounds is not equally distributed among rice varieties (72). Microbial fermentation of rice by-products is an emerging area of scientific and industrial research. Rice bran fermented with *S. cerevisiae* was shown to exert anti-stress and anti-fatigue effects on rats (73). Moreover, water-soluble extracts of fermented rice bran had an anti-photoaging effect on human skin fibroblasts cultures (74). During the last decade, solid-state fungal fermentation of rice bran was extensively studied. The main results achieved concerned the increase of protein content and antioxidant activity (75–77), particularly efficient when the substrate had small particle size (0.18 mm) (78). When defatted rice bran was fermented with *Rhizopus sp*. and *Aspergillus oryzae*, a high amino acids release and consequently a substantial (from 37.5 to 54.3%) chemical score increase (79) were obtained. Apart from proteins, fibers and minerals, rice bran is a good source of oil, which can reach up to 20% of its weight (70). Fermentation with either *Rhizopus oryzae* or *A. oryzae* significantly increased palmitic and linoleic acids content, causing a decrease in saturated fatty acids and an increase in the unsaturated ones (80, 81), thus improving the overall nutritional quality; additionally, when *R. oryzae* was used as starter for rice bran fermentation, a 10% reduction of total lipid content was also observed (80).

Several studies investigated rice bran anticancer properties. Rice bran fermentation with *Lentinus edodes* allowed the production of an exo-biopolymer, consisting of sugars (mainly arabinose, galactose, glucose, mannose, and xylose), uronic acid and a small amount of proteins. Oral administration of the polysaccharide extract induced the activation of natural killer cells and prolonged the life spans of mice transplanted with Sarcoma-180 cells, while inhibiting cancerous cells growth in the intraperitoneum (82). Metabolite formation from extracts of rice bran fermented with *Saccharomyces boulardii* were studied by Ryan et al. (71). It was indeed observed that fermentation altered bioactive compounds, reducing the growth of human B lymphomas *in vitro*. Another study showed that the extract obtained by co-fermentation of rice bran with *Lactobacillus rhamnosus* and *S. cerevisiae*, was able to reduce the cytotoxicity and inhibit melanogenesis in B16F1 melanoma through downregulation of microphthalmia-associated transcription factor (83). Anticancer properties were also found in brown rice bran fermented with *Aspergillus oryzae*. It was demonstrated that fermented rice bran acts as preventive agent against colon carcinogenesis in rats (84).

The antioxidant potential of fermented rice bran was also studied. Phenolic compounds are present in rice bran at high concentrations, however, 70% of them are esterified to the arabinoxylans present in the bran cell wall. Rice bran fermented by *Issatchenkia orientalis*, a yeast isolated from rice bran, showed higher free phenolic content compared to the native one. The extracts strongly inhibited reactive oxygen species generation and ameliorated oxidative stress-induced insulin resistance by neutralizing free radicals and upregulating adiponectin in adipocytes (85). Phenolic extracts obtained by solid-state fermentation of rice bran with *Rhizopus oryzae*, were evaluated for their ability to reduce free radical DPPH and inhibit the enzymes peroxidase and polyphenol oxidase (86). Compared to the native bran, the phenolic content doubled with fermentation and changed in composition, in fact, ferulic acid increased over 20-fold reaching 765 mg/g in fermented bran. Although no inhibition of the polyphenol oxidase enzyme was found, the phenolic extracts DPPH scavenging and peroxidase inhibitory activities (86). The release of phenolic compounds from the bran was also obtained by fermenting heat-stabilized defatted bran with *Bacillus subtilis* subsp. *subtilis*. Compared to the control, which only had low levels of *p*-cumaric and ferulic acids, fermentation allowed the release of gentistic, caffeic, syringic, p-coumaric, ferulic, sinapic, and benzoic acids (87). The potential of fungal solid-state fermentation on the release of bioactive compound having antioxidant activity was investigated (88). Changes in the phenolic profile by *Rhizopus oligosporus* and *Monascus purpureus* fermentation used as single or mixed starters were observed. Although total polyphenols content and ferric reducing ability of plasma increased upon fermentation, DPPH radical-scavenging activity decreased in some cases, due to a different composition in phenolic acids. Ferulic acid was the only phenolic acid present in all samples, before and after fermentation, whereas vanillic, caffeic, and 4-hydroxybenzoic acids were found only after fermentation. The use of the two fungi combined was the condition that allowed the highest release of ferulic acid (almost 8-fold higher than the unfermented bran) (88).

The other by-product of rice milling is the germ, commonly separated by sieving and vibrating rice bran (68). The literature on rice germ is very limited, and studies on fermentation of rice germ initiated only few years ago. Extracts from rice germ fermented by the GABA-producing *Lactobacillus sakei* B2-16 accumulated 15% (of dry weight) of GABA and were found to have a positive impact on sleep disturbance in mice (89).

Regarding their use as food ingredients, the literature is very meager. Fermented rice bran was used in bread-making with the aim of balancing the lacking essential amino acids and enriching the protein content of wheat-based products. The authors evaluated the effect of different substitution levels of wheat with protein concentrates from natural and yeast fermented rice bran. At 10% substitution level, the composite bread had higher total amino acid content than control wheat bread. An increase in the radical scavenging activity and ferric reducing ability power were also observed (90).

### Milling By-Products From Other Cereals

Barley and oat significantly differ in their chemical composition from other cereals; their cell walls are rich in the non-starchy polysaccharide β-glucan, which is the major component of the soluble dietary fiber, and has been associated with the reduction of plasma cholesterol and glycemic index, and a decreased risk of colon cancer (91). Despite the beneficial advantages deriving from the consumption of barley and oat dietary fibers, very little information in the literature deals with the fermentation of their by-products. Catechin and proanthocyanidins are among the polyphenol compounds contained in barley bran. Hordeumin, an anthocyanidin-tannin purple pigment produced from barley bran fermented using *Salmonella typhimurium*, was found to have antimutagen properties (92). Barley bran hydrolysates were used to obtain xylitol through bioconversion of xylose-containing solutions by the yeast *Debaryomyces hansenii* under microaerophilic conditions (93). Xylitol is employed in the food industry to manufacture sugar-free products because of its high sweetening power, anticaries properties, and its tolerance by diabetics (94).

Fermentation was used as means to enrich oat bran with folate. Folate is a generic name for several derivatives of pteroylglutamic acid (folic acid) and is necessary for methylation reactions in cell metabolism and for neural development of fetus during pregnancy (95). Oat bran was fermented with yeasts isolated from barley kernels and selected for the ability to synthesize folate, alone or together with lactic acid bacteria isolated from oat bran. The best folate producers were *S. cerevisiae*, followed by *Pseudozyma* sp., *Rhodotorula glutinis*, and *Kluyveromyces marxianus*. Many yeasts, beyond the considerable amount of folate produced, caused a decrease in the viscosity, suggesting a possible generation of soluble fibers, with positive repercussion on the nutritional effect. When inoculated together with *Streptococcus thermophilus* or *L. rhamnosus, S. cerevisiae* and *Candida milleri* produced significant amount of folates reaching 120 ng/g, suggesting that the consumption of 100 g of fermented oat bran could represents 15% of the recommended folates daily intake (95). Fermentation of oat bran with rye sourdough, previously obtained with a commercial starter culture containing lactobacilli and *Candida milleri*, allowed to double protein and β-glucan solubility (96). Since oat β-glucan can stimulate the growth of *L. rhamnosus*, a fermented oat bran suspension was used as a carrier for the probiotic strain. A simulator of the human intestinal microbial ecosystem (SHIME) was used to evaluate the effect on gut microbiota, concluding that *Lb. rhamnosus* colonized the SHIME and oat bran favored the growth of bifidobacteria (97).

*Lactobacillus plantarum* T6B10 and *Weissella confusa* BAN8 were used as selected starters to ferment maize milling by-products mixtures made with raw or heat-treated germ and bran (98). Lactic acid bacteria metabolisms improved the free amino acids and peptides concentrations as well as the antioxidant activity and induced phytic acid degradation. As previously reported for wheat germ (46), fermentation allowed the decrease of the endogenous lipase activity, stabilizing the matrix by preventing oxidative processes. When fermented maize by-products were used as ingredient for bread making (25% on total weight) dietary fiber and proteins content were of ca. 11% and 13% of dry matter, respectively. Compared to the use of the same amount of unfermented ones, the addition of pre-fermented maize by-products to bread caused a significant increase in protein digestibility (up to 60%), and a relevant decrease of the starch hydrolysis index (ca. 13%) (98).

## Fermentation of the Cereal Industry Waste

### Brewers' Spent Grain

Brewers' spent grain (BSG) is recovered from mashing, one of the initial steps of brewing. During the boiling process, all soluble matter is extracted into the mash from the barley malt which, after lautering (or mash filtration), is separated into wort (liquid) and spent grain (solid) components. BSG represents 85% of the total residues from the brewing process and it is estimated that 30,000 tons of BSG are wasted yearly worldwide. The main current destination of BSG is cattle feed or discarded (10). BSG is rich in cellulose (17%) and non-cellulosic polysaccharides, especially arabinoxylans from the barley grain hull (39%) (99). BSG contains up to 20% of proteins, particularly rich in lysine and approximately 30% of the total protein content is made of essential amino acids (100). Apart from uses in animal nutrition or recovery of valuable compounds such as carbohydrates, proteins and phenolic compounds (101), thanks to the health benefits associated with BSG ingestion, some attempts to exploit its use in food industry have also been made. The research so far has shown that due to BSG challenging technological properties, pre-treatments aiming at reducing its detrimental impact on food quality are necessary (101). Overall, milling and bioprocessing technologies, including fermentation, are potential means to use more of the BSG in food applications, conferring important health benefits. In one study, BSG was used as substrate to immobilize *Lactobacillus casei* suggesting that BSG can act as prebiotic, stimulating lactic acid bacteria growth (102).

A fermented liquid product from BSG having nutraceuticals properties was also developed. After a first substrate optimization phase aiming at improving bacterial growth and polyphenolic compounds release, *L. plantarum* ATCC 8014 was used to produce a fermented beverage displaying high antioxidant potential, due to the high content of total phenolic compounds and flavonoids released during the fermentation, as consequence of the acidification and microbial metabolism (103). Bread containing BSG fermented with a strain of *L. plantarum*, was positively judged for the main structural and sensory properties. Thanks to the high content of proteins, fibers and lysine, a 10% replacement of wheat flour with either BSG or fermented BSG, improved the nutritional properties of the resulting bread (104). Spontaneous fermentation of BSG also showed positive impact on bread properties, characterized by lower levels of phytic acid (more than 30% lower compared to the native counterpart) and higher antioxidant activity (up to 36%) compared to the bread containing unfermented BSG (105).

### Other Cereal-Derived Waste: Bread and Starch

On a global scale, one of the major food waste is bread, reaching thousands of tons daily. Industrial bread waste is generated at different stages: during the manufacturing process, because of substandard products or other processing factors such as crusts removal for sandwich bread production, or as unsold bread from retail (106). If not discarded or used as feed, over the last decades, wasted bread was used to produce chemicals, aroma compounds, enzymes and biofuels (106). The processes to produce a seasoning sauce from the hydrolysis of wheat bread or to produce a syrup from bioprocessed bread were even patented (107, 108). The first attempt involving lactic acid bacteria fermentation was proposed a couple of decades ago. In this study, more than one hundred starters were screened based on the ability to acidify a medium containing bread crumbs. Three strains of *L. plantarum, Staphylococcus carnosus, Pediococcus acidilactici* and *Micrococcus* spp. were selected as able to resist for long fermentation periods (48–96 h) without producing off-flavors (109). A sourdough containing bread waste was produced with the aim of enhancing bread aroma and flavor. Fermentation with a commercial *L. plantarum* strain as starter (35°C for 48 h) of a dough made of 50% whole wheat bread crumb, favored the highest organic acids production (109). Although no specific nutritional features have been highlighted by the authors for the fermented bread, the role of the organic acids, the main responsible of reduced glycaemia and insulinemic responses, should be considered. Indeed, lactic acid lowers the rate of starch digestion in bread whereas acetic and propionic acids appear instead to prolong the gastric emptying rate (110). Therefore, it is assumable that fermented bread can have similar features of sourdough.

Starch production industry mostly interests cereals such as maize, rice, and wheat, and crops like potato, and like any other industry, has its own by-products. Broken rice, which is an inevitable by-product of rice milling, can be used to extract powder and crystal starch generating another by-product rich in proteins (111). The protein residue of starch extraction was treated with a combination of enzyme hydrolysis and microbial fermentation. The hydrolysate resulting from the action of proteolytic enzyme and a cell suspension of *Bacillus pumilus* AG1 displayed antioxidant activity toward ABTS radical. Almost all the peptides contained in the hydrolysate showed one or more features typical of well-known antioxidant peptides, most probably conferring a synergic antioxidant effect to the mixture with the potential to be used as functional ingredient for novel food formulations (112).

### Trends and Perspectives

The future bio-economy concept, based on a more sustainable use of agricultural by-products, will require a more efficient utilization of side streams and waste from food processing industry to reduce the environmental burden of their generation and disposal. The exploitation of cereal by-products for the extraction of their functional compounds, whether for food, cosmetics, or pharmaceutical industry, offers promising alternative to synthetic compounds and it is an increasing trend. Nevertheless, this approach implies that more by-products will be generated once the specific compound is extracted. Furthermore, if the generation of by-products from food industry is unavoidable, the best possible valorization of these by-products should be sought which, in the case of by-products still fit for human consumption, as described in this review, implies their re-utilization within the food chain.

As recently pointed out by the EAT Lancet report^1^, a diet rich in plant-derived food and less relying on animal derived foods is the most beneficial for human health and environment^1^. In this context, the use of the fiber and protein rich part of cereal by-products in food formulations represents a very good opportunity to enrich our diet with beneficial compounds. To contribute to the above objective, the development of technologies allowing the use of the whole by-product, without the undesirable features and with improved nutritional quality is a crucial step.

By exerting an impact on the nutritional properties and potential health effects of bran, germ and all the by-products of the cereal industry, fermentation technology well-responds to the challenge of turning poorly utilized waste into products of interest (Table 1). Increased minerals and vitamins bioavailability, protein content and digestibility, fiber and phenolic compounds solubility are the most common aspects fermentation acts on. However, in some cases other effects have been found, such as the production of bioactive compounds with anti-cancer properties *in vitro*. Some of the nutritional and functional properties of these by-products are well-known, others less, and despite the need and the increased awareness of the impact of diet on health, sensory and organoleptic properties remain the main drivers of consumers' choice. Unfortunately, health benefits and good taste or even appearance, do not always go together; thus, the modulation of processing parameters is required to reach a balance between desired and undesired features. Therefore, more research on the supplementation of these by-products, fermented or as such is needed not only to fill the current technological gaps but also to validate with *in vivo* studies the benefits found *in vitro*: the potential outcome of this approach is worth to be explored further.

**Table 1:** Main nutritional and functional effects of the use of fermentation in cereal industry by-products.

**Cereal by-product**	**Bioprocessing employed**	**Effect**	**References**
Wheat bran	*Lb. brevis* E95612 and *K. exigua* C81116 with enzymes; baker's yeast; spontaneous fermentation; *Lb. bulgaricus* and *St. thermophilus* combined with baker's yeast	Higher fiber solubility	(24–26, 59)
	*Lb. brevis* E-95612 and *Candida humilis* E-96250 with cell wall-degrading enzymes; *Lb. brevis* E95612 and *K. exigua* C81116 with enzymes; spontaneous fermentation	Increased peptides and free amino acids content and *in vitro* protein digestibility	(24, 25, 29)
	Spontaneous fermentation; baker's yeast; *Lb. bulgaricus* and *St. thermophilus* combined with baker's yeast	Decreased phytic acid content	(25, 26, 35)
	Baker's yeast; *A. oryzae* MTCC 3107; *Hericium erinaceus;* spontaneous fermentation; lactic acid bacteria and yeasts with enzymes	Higher phenols content and antioxidant activity	(25, 31–34)
	*Propionibacterium freudenreichii* DSM 20271	Fortification in vitamins	(38)
	*Mucor* spp.	Increase of gamma-linolenic acid and β-carotene content	(64)
Wheat germ	*Lb. plantarum* LB1 and *Lb. rossiae* LB5	Increased free amino acids content, protein and minerals bioavailability, decreased anti nutritional factors	(46)
	*B. subtilis* B1; *Lb. plantarum* LB1 and *Lb. rossiae* LB5	Increased antioxidant activity due to phenolics or bioactive peptides	(46, 49)
	*S. cerevisiae*; *Lb. plantarum* LB1 and *Lb. rossiae* LB5	*In vitro* and *ex vivo* anticancer and antiproliferative properties	(50–53)
Rye bran	Baker's yeasts	Release of phenolic compounds, increase of folates content	(59)
	*Lactobacillus reuteri*; *Weissella confusa*	Exopolysaccarides synthesis	(65, 66)
	*Mucor* spp.	Increase of gamma-linolenic acid and β-carotene content	(64)
Rice bran	*S. cerevisiae*	Anti-stress, anti-fatigue and anti-photoaging effect	(73, 74)
	*Aspergillus oryzae; Rhizopus oryzae*; *Rhizopus oligosporus and Monascus purpureus; Lb. rhamnosus* and *S. cerevisiae*;	Higher protein and phenols content and antioxidant activity	(75–78, 85–88, 113)
	*Aspergillus oryzae; Rhizopus oryzae*	Decrease of saturated fatty acids and total lipids and increase of unsaturated fatty acids	(80, 81)
	*Lentinus edodes*; *A. oryzae*; *S. boulardii*; *Issatchenkia orientalis*;	*in vivo* and *ex vivo* anticancer and antiproliferative properties	(71, 82–84)
Rice germ	Spontaneous fermentation	Increased in GABA content and improvement of sleep disturbances in mice	(89)
Barley bran	*Salmonella typhimurium*; *Debaryomyces hansenii*	Production of phenolics and xylitol	(92, 93)
Oat bran	*Streptococcus thermophilus, Lb. rhamnosus, S. cerevisiae* and *C. milleri*	Folic acid fortification	(95)
	*C. milleri*	Higher fiber solubility	(96)
BSG	*Lb. plantarum* ATCC 8014	Higher phenols content and antioxidant activity	(103)
Bread waste	*Lb. plantarum*	Production of a sourdough with high content in organic acids	(109)
Protein from rice starch extraction	*Bacillus pumilus* AG1	Bioactive peptides with antioxidant activity	(112)

## Author Contributions

MV, CR, and RC wrote and critically evaluated the manuscript making substantial, direct and intellectual contribution to the work.

### Conflict of Interest Statement

The authors declare that the research was conducted in the absence of any commercial or financial relationships that could be construed as a potential conflict of interest.

## Footnotes

^1^https://eatforum.org/eat-lancet-commission/

## Abbreviations

ABTS, 2: 20-azino-di-[3-ethylbenzthiazoline sulphonate]
BSG: Brewers' Spent Grain
DPPH, 2: 2-diphenyl-1-picrylhydrazyl
GABA: γ-aminobutyrric amino acid
SHIME: Simulator of the Human Intestinal Microbial Ecosystem.
